# Supplementary material for: Iterative improvement in the automatic modular design of robot swarms
Source: PeerJ Comput Sci. 2020 Dec 7;6:e322. doi: 10.7717/peerj-cs.322 (PMC7924708; doi:10.7717/peerj-cs.322)
Supplement: Supplemental Information 3 [file peerj-cs-06-322-s003.zip › argos3/doc/api/standalone/a00634_source.html]

ARGoS: plugins/simulator/visualizations/qt-opengl/models/qtopengl\_light.h Source File


- Main Page
- Related Pages
- Namespaces
- Classes
- Files

- File List
- File Members

# plugins/simulator/visualizations/qt-opengl/models/qtopengl\_light.h

Go to the documentation of this file.

```
00001 
00007 #ifndef QTOPENGL_LIGHT_H
00008 #define QTOPENGL_LIGHT_H
00009 
00010 namespace argos {
00011    class CQTOpenGLLight;
00012    class CLightEntity;
00013 }
00014 
00015 #ifdef __APPLE__
00016 #include <gl.h>
00017 #else
00018 #include <GL/gl.h>
00019 #endif
00020 
00021 namespace argos {
00022 
00023    class CQTOpenGLLight {
00024 
00025    public:
00026 
00027       CQTOpenGLLight();
00028 
00029       virtual ~CQTOpenGLLight();
00030 
00031       virtual void Draw(CLightEntity& c_entity);
00032 
00033    private:
00034 
00035       GLuint m_unList;
00036       GLuint m_unVertexes;
00037       GLfloat m_fRadius;
00038 
00039    };
00040 
00041 }
00042 
00043 #endif
```

---

Generated on 10 Jul 2018 for ARGoS by 
 1.6.1
